# Digital assistance systems in the field of incontinence care for individuals in need of long-term care (EASY): study protocol of a stratified randomised controlled trial

**DOI:** 10.1186/s12877-023-04135-2

**Published:** 2023-07-05

**Authors:** Stefan Schmidt, Alexandra Neumann, Julie Muller, Anja Schweitzer, Katharina Ingrid Gölly, Julio Brandl

**Affiliations:** 1 Faculty of Health, Nursing, Management, University of Applied Sciences Neubrandenburg, Brodaer Strasse 2, Neubrandenburg, 17033 Germany; 2 AssistMe GmbH, Bachstrasse 12, Berlin, 10555 Germany

**Keywords:** Incontinence, Stratified randomised controlled trial, Digital assistance system, Sensor, Fall prevention, Sleep quality, Quality of life, Smartphone, Care

## Abstract

**Background:**

In general, urinary and faecal incontinence is a multifaceted problem that is associated with increasing burdens for those affected, a massive impairment of quality of life and economic consequences. Incontinence is associated with a high level of shame, which in particular reduces the self-esteem of those being incontinent and leads to additional vulnerability. Those affected by incontinence often perceive incontinence and the care they receiveas humiliating, hence they can no longer control their own urination; nursing care and cleansing support then lead to additional dependency. People with incontinence in need of care not uncommonly experience a poor communication and many taboos surrounding the issue as well as the use of force when incontinence products are changed.

**Aims and methods:**

This RCT aims to validate the benefits of using a digital assistance system to optimise incontinence care and to enable statements concerning the effect of the assistance technology on nursing and social structures and processes as well as on the quality of life from the perspective of the person in need of care. In a two-arm, stratified, randomised, controlled interventional study, primarily incontinence-affected residents of four inpatient nursing facilities will be examined (*n* = 80). One intervention group will be equipped with a sensor-based digital assistance system, which will transmit care-related information to the nursing staff (via smartphone). The collected data will be compared to the data of the control group. Primary endpoints are falls occurring; secondary endpoints are quality of life and sleep, sleep disturbances and material consumption. In addition, nursing staff (*n* = 15–20) will be interviewed regarding the effects, experience, acceptance and satisfaction.

**Discussion:**

The RCT aims at the applicability and effect of assistance technologies on nursing structures and processes. It is assumed that, amongst other things, this technology may lead to a reduction of unnecessary checks and material changes, an improvement of life quality, an avoidance of sleep disturbances and thus an improvement of sleep quality as well as to a reduced risk of falling for people with incontinence in need of care. The further development of incontinence care systems is of social interest as this offers the prospect of improving the quality of care for nursing home residents with incontinence.

**Trail registration:**

Approval of the RCT is granted by the Ethics Committee at the University of Applied Sciences Neubrandenburg (Reg.-Nr.: HSNB/190/22). This RCT is registered at the German Clinical Trials Register on July 8^th^, 2022, under the identification number DRKS00029635.

## Background

Incontinence refers to the body’s inability to retain and release urine (urinary incontinence) or faeces (faecal incontinence) in a controlled manner, resulting in an involuntary outflow. Experts estimate that between six and eight million people in Germany are affected by urinary incontinence and about four million are affected by faecal incontinence[1–3]. The exact prevalence of urinary and faecal incontinence is difficult to determine due to the different care settings (inpatient and outpatient) and data collection methods. Incontinence is often associated with further health problems such as dermatitis, falls, reduced daily activity, longer hospitalisations and higher mortality, whereby the overall quality of life of those affected is severely effected [4–7]. In addition, individuals affected by incontinence often experience psychological impairments such as a lack of dignity [8] because they can no longer control their own urination and defecation; care and cleansing support then lead to additional dependency [9]. If support is needed as part of care, those affected not uncommonly experience a poor communication and taboos concerning the issue as part of the everyday routine nursing care and even the use of violence during the change of incontinence material [10]. As the prevalence of incontinence increases with age this is predominantly a problem for long-term care [11]. It is estimated that approximately 80.4% of the individuals in long-term care are affected [1], and incontinence is most prevalent in individuals with increased care needs [12, 13]. If individuals are no longer able to express for themselves that incontinence products need to be changed, the nursing staff make these decisions. Whereby this leads to unnecessary controls of incontinence material, which are however often unavoidable and are accompanied by a disturbance of the person in need of care. In particular, nightly changes of incontinence materials have a negative impact on those affected, as additional sleep interruptions further reduce the already low quality of sleep [14]. The often unnecessary checks and changes of incontinence materials also have economic consequences. When calculating these effects, direct (e.g. costs for incontinence materials), indirect (e.g., loss of resources) and intangible costs (e.g. reduction in quality of life, restrictions on individual freedom) are differentiated, with direct costs being the simplest to calculate. A study conducted in 2007 analysed that the direct costs of urinary incontinence in Germany amounts to approximately €396 million. 70% of these costs (€277 million) are attributed to women and 30% (€118.6 million) are attributed to men. [15]. The direct costs of incontinence care are thus comparable to those of other chronic diseases such as dementia and diabetes [16, 17]. The indirect and intangible costs have not been scientifically evaluated yet but are estimated to be higher than the direct costs. Since the costs are significantly related to the severity of the incontinence as well as to the age and degree of care required by the affected individuals, the demographic change, amongst other factors, ensures that the costs will double in the next 50 years in Germany [18]. Despite the high prevalence of incontinence in long-term care [19], few international and national studies can be identified to date that adopt a nursing science perspective, revealing a research gap. Most studies focus on specific diseases and medical parameters but neglect nursing aspects, or they focus on physiological endpoints such as the effects of pelvic floor muscle and bladder function training (e.g. [20–24]. Studies with technical interventions in the field of incontinence are scarce and, if available, they focus tightly on medical parameters [25] or their results cannot be transferred to the primary target group of long-term care residents [26, 27]. Up to now there are only a few studies focusing on nursing parameters [among others, [27, 28]. Studies on the use of digital assistance systems in incontinence care with people in need of care that investigate the consequences on quality of life and sleep as well as the benefits of the technology on the quality of care overall have not been conducted yet. However, the combination of sensor technology with technical assistance systems in the field of incontinence care has made a significant progress in the past few years. (e.g. the system from AssistMe GmbH).

Routine visual inspections of care recipients' incontinence products are currently performed as standard practice in both outpatient and inpatient settings. This procedure significantly compromises a variety of aspects such as hygiene, ethics, the structure of daily life and work efficiency. A digital, sensor-based assistance system could replace visual checks in both outpatient and inpatient settings and improve the quality of incontinence care for both those in need of care as well as for nursing staff. Although the benefits of digital assistance systems in the field of incontinence care are promising, primarily the users (e.g. family caregivers, nursing staff) need to be convinced. Technical aids and digital assistance systems often lead to scepticism among nursing staff, this manifests itself in misunderstandings, worries and fears, a lack of information and insecurities concerning data usage [29]. This skepticism could be countered by letting nursing staff experience the benefits of these systems (e.g., improvement of incontinence care, avoidance of unnecessary changes) and by creating a familiarity with the assistance system among all participants. The latter is particularly the case when users are involved in the development of technical innovations. This also reduces the risk that technical aids are developed "parallel" to the care process [29]. Studies on this topic have not been conducted yet.

## Objectives and questions

This RCT aims to validate the benefits of using a digital assistance system to optimise incontinence care and to enable statements concerning the effect of assistance technology on nursing and social structures and processes as well as on the quality of life from the perspective of individuals in need of care. Answers are to be found in particular to the following questions:

- What are the benefits of planned assistance technologies for incontinent care recipients?
- Can assistance technologies reduce falls and improve quality of life as well as quality of sleep of incontinent individuals?
- What effects does the assistance technology have on existing structures and care processes in long-term care?
- How can implementation in nursing practice be organized and what kind of logistical measures are required for usage in long-term care (such as information materials, training of nursing staff, maintenance and care of the technical aids to ensure the required accuracy and reliability, procurement/disposal)? How can the technology be incorporated into routine processes in long-term care?
- In terms of economic perspectives, what results does a measurement or prognosis of success provide to potential users of assistance technologies?
- How can a sustainable utilisation of the technologies be assured?

In the long term this project aims to contribute to new quality-assured modes of care in nursing practice.

## Methods and design

This RCT is based on a mixed-methods approach and consists of a quantitative and a qualitative method strand, which are to be arranged in a parallel design to each other, allowing an integrative presentation of the results. The focus is on individuals requiring care in inpatient nursing facilities (primary target group) and on nursing staff (secondary target group). The approach selected intends to generate a comprehensive understanding concerning the use of digital assistance systems for incontinence care, which represents the different perspectives of the target groups and captures the complexity of the research subject as a whole.

This study protocol is guided by the SPIRIT Statement [30].

**Fig. 1:**
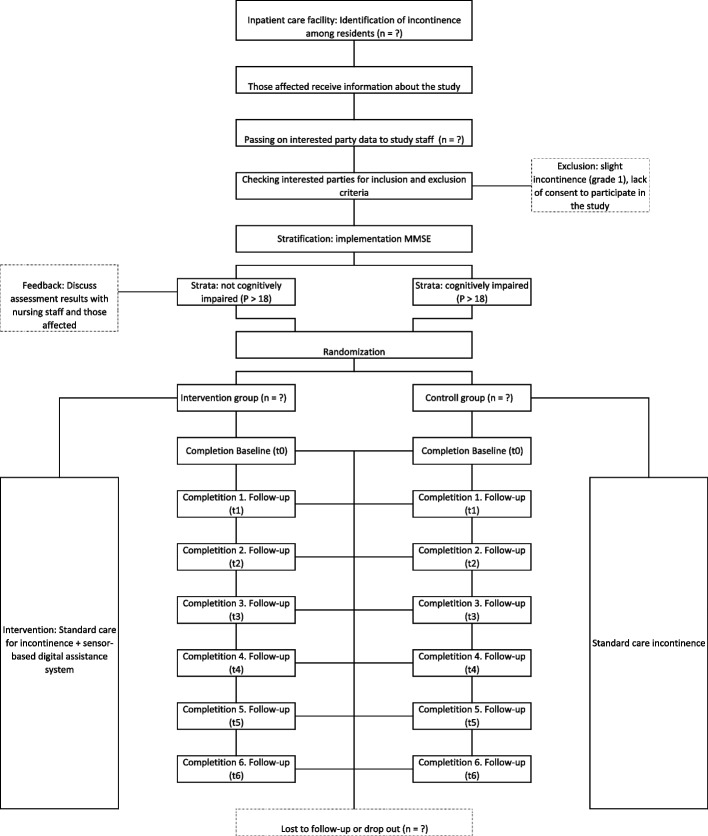
Flow chart concerning the primary target group [37]

Concerning the *primary target group of care recipients*, a two-arm, stratified and randomised, controlled interventional study is selected for the study design (RCT). Participants will be assigned to the intervention group (IG, equipped with a digital assist system) and control group (CG, not equipped with a digital assist system) study arms, and the assignment of study participants to arms will be randomized. To ensure a homogeneous distribution regarding the prognostic factor of impaired abilities to the IG and CG, a simple disproportionate stratification will be performed prior to the randomisation. This will ensure that a valid interpretation on the efficacy of the intervention is possible. Other advantages of this approach refer to a reduced probability of an α and/or β error when interpreting the results, furthermore the statistical validity of the results with a comparably low participant number is increased, and interim analyses are simplified [31–33]. The participants will be stratified using the Mini Mental Status Examination (MMSE). On the basis of the points achieved in the questionnaire, care recipients will be allocated to the subgroups “cognitively impaired” (points < 18) and “not cognitively impaired” (points > 18). In previous studies, the use of self-reported scales up to an MMSE point value of > 10 proved to be appropriate [including [34, 35]. However, in the study implementation, the results of the MMSE are used for stratification from a score of > 18. As a result, the RCT is based on the original values of the instrument [36]. Participants that have been identified as cognitively impaired by the assessment results of the MMSE (score 0 to 18) will be reported back: both nursing staff and participants affected shall estimate as objectively as possible whether the benefit of self-reported scales is appropriate. Participants who score below 18 on the MMSE or for whom assessment feedback argues against self-assessment scales will be administered assessments as third-party assessment scales.. After stratification, the participants of the two subgroups will be randomized individually to the two study arms IG or CG (see Fig. 1).

Concerning the *secondary target group of nursing staff*, a single-armed observational study design is selected. All participants will be surveyed both quantitatively and qualitatively at four time points.

When randomizing both groups, additional attention will be paid to the individual framework conditions of the participating inpatient care facilities and they will be appropriately responded to.

## Methods

Within the framework of the RCT, care recipients affected by incontinence and their responsible nursing staff will be monitored in four selected inpatient care facilities in Berlin and Brandenburg over a system test period of four months.

### Inclusion criteria

#### Primary target group

To consider the different comorbidities of the primary target group and to be able to realistically assess the benefits of the digital assistance system, the inclusion criteria were defined relatively broadly. Individuals included in the RCT:

- have a moderate to severe incontinence (degree of severity ≥ 2, urinary or urinary and faecal incontinence) and
- have given their written informed consent to participate in the RCT after receiving information and clarification. If individuals are not able to consent to study participation by themself due to cognitive impairments, their family caregivers or legal guardians may provide consent.

The severity of incontinence is determined by the consumption of incontinence material per person in need of care as recorded in the care documentation.

Due to the multifaceted occurrence of incontinence, other factors such as cognitive ability, mobility as well as the degree of care of the person affected do not play a role as criteria for study participation. Individuals who are cognitively impaired as well as not impaired; who are mobile as well as immobile; and who have a high or low level of care will be included in the RCT.

#### Secondary target group

Since nursing staff is to incorporate and evaluate the digital assistance system as part of their professional activities, only the nursing staff responsible for incontinence care is included in the study. By doing so, differentiated statements about the system can be voiced by the nursing staff. Ideally, the included nursing staff is also responsible for participants in the control group, enabling them to identify and describe any changes concerning nursing care with an even greater nuance. Furthermore, a written consent to participate in the study after being informed and educated is necessary. In addition, nursing staff with similar as well as different characteristics such as professional experience, age and gender is included. Thereby, the individuals should differ as minimally and maximally as possible. This contrastive sampling procedure intendeds to reflect the heterogeneity of the research subject through the selection of individuals to be interviewed [38].

### Exclusion criteria and dropout management

#### Exclusion criteria of the primary target group

In order to include as many individuals as possible in the RCT, the exclusion criteria will be defined relatively narrow.

Individuals will be excluded from the RCT if:

- they or their responsible nursing staff decline to participate in the study and therefore do not sign the written declaration of informed consent, and if
- they only have a mild form of incontinence (degree of severity 1, urinary or urinary and faecal incontinence).

#### Exclusion criteria of the secondary target group

Nursing staff will only be excluded from the study if they do not agree in writing to participate and/or if they are not responsible for the incontinence care of the primary target group.

#### Dropout criteria of the primary target group

Individuals may withdraw from their study participation at any time. They or their responsible nursing staff can revoke their consent to participate in writing. Likewise, they are automatically excluded from the RCT if they die during the intervention.

A deterioration in their health does not necessarily lead to exclusion from the RCT. Those affected are only excluded from further study events if the deterioration is due to compelling medical and/or nursing reasons requiring a termination of their participation in the study.

#### Dropout criteria of the secondary target group

Nursing staff may withdraw their consent to participate at any time. They can do this by revoking their consent to participate in writing.

In addition, nursing staff can be excluded from the study if their poor technical affinity or acceptance leads to massive restrictions concerning the correct implementation and use of the digital assistance system in the context of incontinence care. In order to ensure a correct application of the technology, training sessions for the nursing staff will be held over several days at the beginning of the measurement period. During this training nursing staff will be introduced in depth to the digital assistance system. This is followed by a test phase of one to two weeks in the inpatient care facility, during which the nursing staff independently tests the system in regular care. If, during this period, a professional is found to lack technical user skills or feedback is received from the person concerned, this person will not continue to participate in the study.

For both the primary and secondary target groups, further progression of the Covid pandemic may result in exclusion or withdrawal from the study. If, in an inpatient care facility, a Covid outbreak is deemed by management to have a lasting impact on normal business operations, the facility may decide to not continue its participation in the measurements.

### Interventions

The RCT addresses the use of digital assistance technologies in the field of incontinence care for care recipients. The RCT plans to equip incontinence products of any type (urine and stool) with a sensor that transmits care-related information to connected systems (such as smartphones and PCs). A local tracking mechanism allows the incontinence product to be located, but this function will not be used within this RCT.

The sensor technology can be used within the facilities even without a dedicated W-LAN, as it is used via a Bluetooth Low Energy (BLE) as well as via LTE network; set up by AssistMe GmbH (practice partner). AssistMe provides the complete sensor, hardware and network technology. This also includes the smartphones provided to the nursing staff.

At this stage multiple utilization of the sensors with incontinence material is not possible for the entire course of this study. However, in some cases, particularly at the start of the RCT, sensors will undergo a re-use process (including cleaning, disinfection, etc.) to check and test if and to what extent multiple utilization is possible. In view of the above, a multiple utilization of the sensors will be aimed at for test purposes, in order to identify opportunities for cost-effective mass production while also ensuring a sustainable use of the product. The project in applied practice thus enables further investigations which can provide recommendations for mass production of incontinence products equipped with (printed) sensors. The experience of the practical partner AssistMe GmbH in the field of cleaning and disinfecting sensor technology will also be used for quality assurance and development of logistical recommendations.

The digital assistance system from AssistMe GmbH provides the responsible nursing staff within the care facilities with an automated message concerning the status of the incontinence material (ICM) of the home residents within the IG. The status report does not give any indication about whether the ICM needs to be changed but only describes the status of the ICM itself (for example, 1st urination, 2nd urination, 3rd urination). The decision to change the ICM is made by the responsible nursing staff and not by the digital assistance system from AssistMe GmbH. In addition, it is possible to store individual incontinence profiles of the home residents in order to obtain an additional source of information concerning the decision among the nursing staff as to when an optimal change of the ICM should take place.

The digital assistance system from AssistMe GmbH is to be understood as a supporting, digital measure in the context of incontinence management.

The technical procedure of the intervention is divided into three phases:

1. Preparation and implementation phase
2. Training and testing phase
3. Realisation phase

- (1) Preparation and implementation phase

This thus implies, on the one hand, the implementation of an IT infrastructure within the care facility and the associated assurance of a complete connectivity of the wearables (explanation, follows below) equipped to the ICM with the network. On the other hand, the logistical process of the ICM requirements for the home residents will be jointly coordinated in detail with the respective care facilities and service providers (TZMO and geria + med) so that a) no supply bottlenecks arise and b) the study process can be realized as smoothly as possible.

- (2) Training and testing phase

The training and testing phase is used to familiarise the participating nursing staff with the digital assistance system from AssistMe GmbH. This means both a multi-hour training session for nursing staff and a 1–2 week trial period to "get to know" and "better understand" the system will be conducted, in order to reduce any technical challenges during the course of the study and to address any questions that arise from the nursing staff in advance.

- (3) Realisation phase

**Fig. 2:**
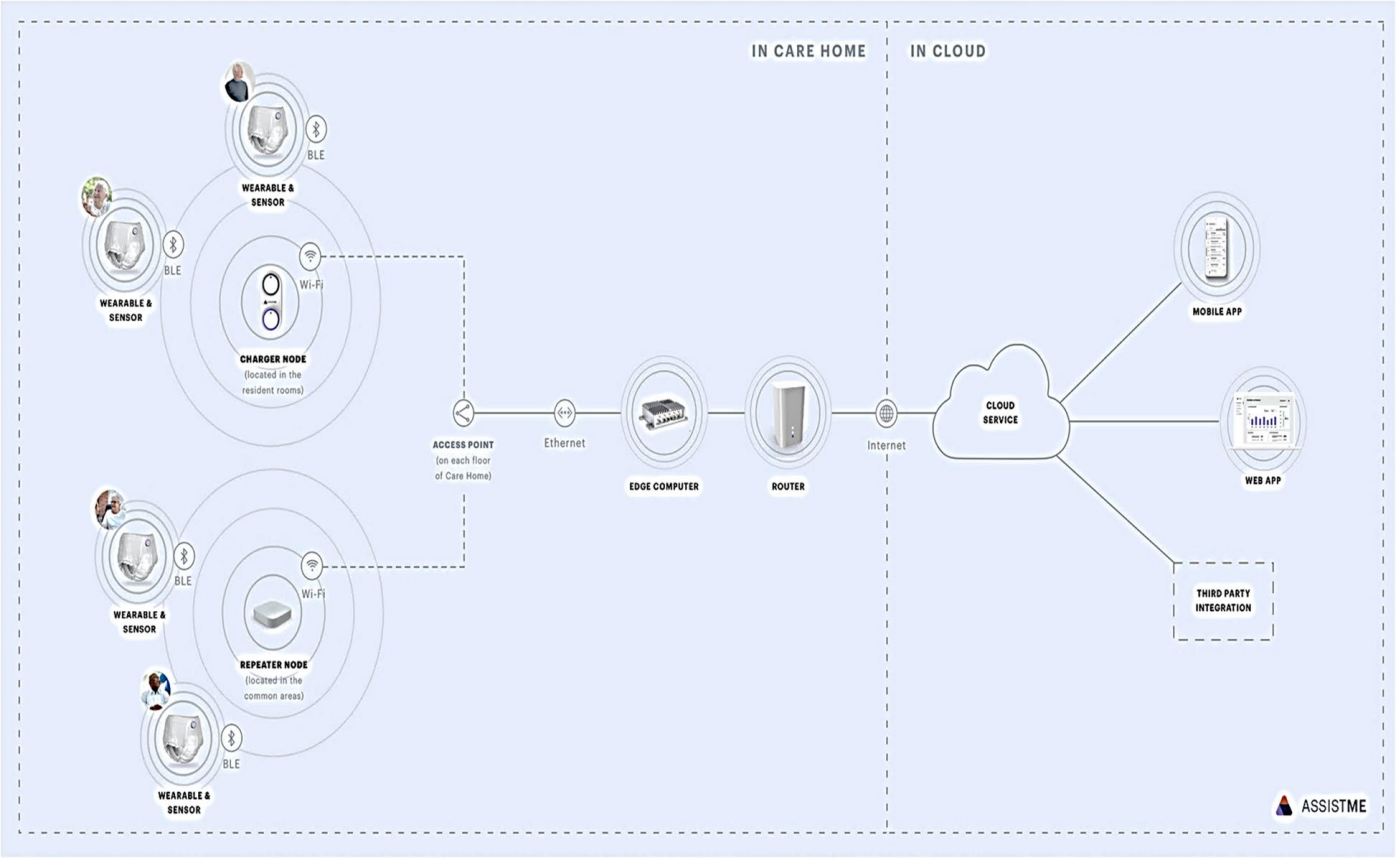
Overview of the digital assistance system from AssistMe GmbH

The realisation phase represents the actual RCT and intervention. In this phase the digital assistance system is connected within the nursing facility, the nursing staff has been trained and the study project begins (see Fig. 2).

The ICM equipped with a sensor is worn with a so called wearable. The wearable records the sensor data and transmits the data via BLE to the corresponding charger or repeater nodes.

The charger and repeater nodes act as network within the nursing facility ensuring a permanent connectivity between wearable and IT infrastructure. The charger and repeater nodes record the signal transmitted via BLE and then forward it via WLAN to defined access points within the nursing facility. These access points send the relevant data to an edge computer in the corresponding nursing facility and then to a LTE router which sends the data to the AWS cloud service, which complies with data protection requirements. The data is processed in the cloud and then forwarded to the mobile application of the responsible nursing staff.

Two wearables will be individually assigned to each resident of the IG and will be accordingly available to the nursing staff. Hence one wearable can be worn with the ICM while the other wearable is charging on the station (charger node) ensuring a seamless change of the wearable when the battery is low.

The aim is to implement a fully functioning end-to-end system in the nursing facility so that the ICM status of each facility resident can be monitored in real time on the smartphone of the responsible nursing staff.

### Endpoints

#### Endpoints of the primary target group (care recipients with urinary and faecal incontinence)

Occurring falls represent the primary endpoint of the RCT. Secondary endpoints include cognitive ability, quality of life, sleep quality and disruptions of sleep, incontinence material consumption and general health.

#### Endpoints of the secondary target group (nursing staff)

The secondary target group will be surveyed by means of interviews regarding the acceptance, experience and perceived effects of the digital assistance systems and the resulting changes to their work processes (secondary endpoints). This qualitative data collection captures the important perspectives of nursing staff and nursing management.

In addition, the nursing staff will also be surveyed quantitatively about their subjective attitude toward the use of technology in their professional context and concnerning the use of the digital assistance system in particular. The subscales of the questionnaire to measure the affinity for technology scale (TA-EG) are used as guidance. The TA-EG consists of 19 items and four scales and has been adequately reviewed in terms of its validity and reliability. Due to its scope, it will be used in a shortened form in the study and adapted to the specific core of the intervention. The modified questionnaire was tested in pretests with regard to comprehensibility, practicability and temporal scope. Subsequently, editorial changes were incorporated (see Table 1).

**Table 1:** Endpoints and measurement tools for the primary target group

Primary endpoints	Measurement tools
Incipient falls	**STRATIFY instrument** (St. Thomas’s risk assessment tool in falling elderly inpatients) [39] • Includes five items / risk factors • Confirmed validity and reliability [40] • Quantitative method, third-party assessment **Tinetti Test** [41] • Includes 17 items • Confirmed validity and reliability [42] • Quantitative method, third-party assessment **Extrinsic and intrinsic risk factors** for the residents • based on and correlating with the national expert standard on incontinence of the German Network for quality standards in care-giving (DNQP) [11] **Analysis of fall records** • Documentation through the nursing facility is mandatory • Provides important information about occurring falls and possible consequences of falls
**Secondary endpoints**	**Measurement tools**
Cognitive ability	**Mini Mental Status Examination (MMSE)** [36] • Consists of five categories with a total of 11 items • Confirmed validity and reliability • Used for stratification • Quantitative method, self-reported scale
Quality of life	**Quality of life in elders with multimorbidity (FLQM**) [43] • Consists of three items • Confirmed validity and reliability • Qualitative and quantitative method, self-reported or third-party assessment scale
Sleep quality and disruptions of sleep	**Karolinska Sleepiness Scale (KSS)** developed by [44], German version based on [45] • Includes 1 item • Not yet validated for older people or geriatric patients • Quantitative method, self-reported or third-party assessment **Richard Campbells Sleep Questionnaire (RCSQ)** • Consists of 5 items • Quantitative method, self-reported or third-party assessment
Consumption of incontinence materials	**Complete documentation** of material usage
General health	Analysis of the nursing documentation for incipient chronic diseases & changes in the severity of existing diseases

### Measurement period

In the context of the intervention study, the incontinence-affected individuals included are accompanied over a system test period of four months. The measurements will take place exclusively in the respective inpatient care facility. For better supervision by the study management, the measurements should be carried out in a maximum of two facilities in parallel during the project period. The measurements will be carried out exclusively by the study staff of the University of Applied Sciences Neubrandenburg.

A total of seven measurement points are planned for the participants of the primary target group. One measurement will take place at the start (t0) and one at the end (t6) of the four-month study period. Between these two points (t0 and t6), the quantitative surveys will be conducted every three weeks (t1, t2, t3, t4, t5). If necessary, when taking the measurements, the study staff of the Neubrandenburg University of Applied Sciences may be assisted by the nursing staff of the relevant inpatient nursing facility (e.g. if a participant has a greater cognitive impairment).

**Fig. 3:**
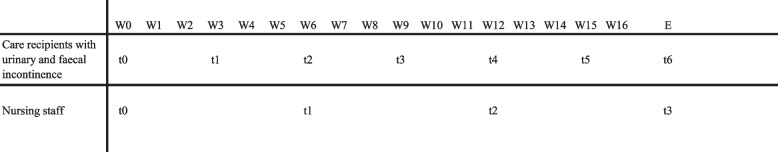
Scheduling of the measurements by target group

Four measurement points are planned for the individuals of the secondary target group. One measurement will take place at the beginning (t0) and one at the end (t3) of the four-month study period. In the meantime, the qualitative and quantitative surveys will take place every six weeks (t1, t2) (see Fig. 3 and Table 2).

**Table 2:** Outcomes, measurement tools and follow-ups [11, 36, 39–43, 46–50]

Primary outcome	Measurement tool	t0	t1	t2	t3	t4	t5	t6
Cognitive ability	MMSE [36]	X						
Incipient falls	STRATIFY [39, 40]	X	X	X	X	X	X	X
	Tinetti Test [41]	X	X	X	X	X	X	X
	Risk factors as per DNQP expert standard [11]	X	X	X	X	X	X	X
	Analysis of fall records [42]	X	X	X	X	X	X	X
**Secondary outcomes**	**Measurement tool**	**t0**	**t1**	**t2**	**t3**	**t4**	**t5**	**t6**
Quality of life	FLQM [43]	X	X	X	X	X	X	X
Sleep quality and disruptions of sleep	KSS	X	X	X	X	X	X	X
	RCSQ [46–50]	X	X	X	X	X	X	X
Consumption of incontinence material	Documentation	X	X	X	X	X	X	X
General health	Nursing documentation	X	X	X	X	X	X	X
Effects	Qualitative interview	X	X	X	X			
Experience	Qualitative interview	X	X	X	X			
Acceptance	Qualitative interview	X	X	X	X			
Changes to workflows	Qualitative interview	X	X	X	X			
Technology affinity	TA-EG (modified)	X	X	X	X			

### Power calculation

The sample size calculation is based on the RCT by Chittrakul et al. [51] in which a special exercise program for fall prevention to avoid falls and increase the quality of life was developed. The result of the RCT was that the participants in the IG fell on average 1.13 times (SD = 0.84) and in the CG the average was 2.22 [51]. For the current sample size calculation, a more conservative estimate was made (M = 1.65) since a less intensive intervention is to be studied that is nevertheless of high clinical relevance. To detect a significant difference between the two groups in a two-arm test with a significance level of 0.05 and 80% power, we need at least 34 persons in each group. Thus, a total of at least 68 persons will be included. Due to the high vulnerability of the sample and assuming a drop-out rate of about 15%, a total of 80 indiviudals will be included with 20 participants from each nursing facility (primary target group). The participants will be divided into an intervention group and a control group.

Concerning the nursing staff, as secondary target group, about six to eight individuals in each inpatient facility, who are responsible for the incontinence care of the participants in the primary target group and routinely use the assistance system in their daily professional practice will be recruited. The sampling of nursing staff follows a qualitative approach and aims to ensure the representativeness of the target group [38] by contrasting characteristics such as gender and professional experience.

### Recruitment

#### Primary target group

The recruitment procedure is divided into two phases: 1) The study staff of the Neubrandenburg University of Applied Sciences and those responsible from AssistMe will visit the participating inpatient nursing facilities. In an open event all residents of the facility who are interested will be informed about the RCT (including the aims of the study, procedure and the intervention). This is meant as opportunity to ask questions to the persons responsible for the RCT. This shall emphasise the absolute voluntary nature of study participation. 2) If the residents or their family caregivers/legal guardians indicate interest in participating in the study, they inform the responsible nursing staff in the facility. The nursing staff consolidate the interested parties and forward their names to the study staff at the Neubrandenburg University of Applied Sciences. The study staff then visits the interested parties again. In a conversation, they will be again informed in detail about the RCT as well as the intervention. In addition, the inclusion and exclusion criteria will be checked. Residents who meet the eligibility criteria and who would like to participate in the RCT are then examined regarding their cognitive performance using the Mini Mental Status Examination (MMSE). Depending on their score, they are assigned to the respective strata "cognitively impaired" or "not cognitively impaired." Subsequently, both subgroups are randomized individually into the intervention and control group.

Should individuals in the primary target group drop out of the RCT prematurely (e.g., due to compelling medical or nursing reasons), new particpipants will be recruited and included if they meet the inclusion criteria. This post-recruitment is foreseen up to a maximum of two months after the start of the system test period.

#### Secondary target group

Only nursing staff responsible for incontinence care of participants in the primary target group within the respective inpatient nursing facility will be recruited. In addition, they must have attended the training sessions concerning the use of the digital assistance system and must have consented to participate in the study.

### Group assignment

The aim is to ensure that IG and CG do not differ beyond the intervention with regard to the prognostic factor of the individuals' cognitive performance. Especially with a small sample size, a different approach could promote an unbalanced relationship between the two groups [31]. An independent researcher, who is not involved in the study, performs the allocation with the software Excel (version 2018) using a randomization list with simple random sampling. During this process, participants are also assigned a code (participant ID), under which they and their data are managed in the further course of the study.

### Blinding

The study information for participants includes information about the assigned arms and the randomised design of the study. An independent researcher not involved in the study performs the allocation using Excel. Participants, nursing staff, the provider of the digital assistance system and research study staff will not be blinded to the group assignment.

### Data management

Data management is divided into two strands. First, data from all survey points, all measurement times and assessment tools (e.g., MMSE) will be documented using electronic case report forms (eCRF). If possible, a plausibility check is performed when the data is first entered into the system. Only the anonymised particpant IDs are used for the eCRFs. Identifying data that enables the allocation of a participant ID to an individual are stored separately. Only the project management has access to this data after random allocation and assignment of the participants IDs. The data is stored on a central network system based on the data management system of the Neubrandenburg University of Applied Sciences in line with current standards for data security. Access to this network system is only granted to study staff at the Neubrandenburg University of Applied Sciences. Access to the system can only be granted by the technical administrator of the Faculty of Health, Nursing, Management after prior agreement. After completion of the study, the identifying data will be stored physically separate in the Neubrandenburg University of Applied Sciences. All study information in paper format, including the written declarations of informed consent and the questionnaires, will be stored in sealed filing cabinets in areas with restricted access. The individuals included in the study provide a written declaration of informed consent based on comprehensive verbal and written information provided about the study. All consent forms will be stored in the Neubrandenburg University of Applied Sciences. If a study participant limits his or her consent, the limitation is documented and the subsequent data analysis is performed in accordance with the imposed limitations. If a person withdraws his or her consent, all data will be removed, with the exception of data that was published prior to the withdrawal of the consent. All data will be analysed only in an anonymised format. For publications, only aggregate data that does not permit personal identification will be used. Data will only be shared without identification of the participants. Data management and the documentation process will be monitored by the study management.

The second part of the data management covers data collected during the study intervention. The data transfer between the individual components of the digital assistance system (wearable, node, edge computer, cloud) is end-to-end encrypted using a high security standard. End-to-end encryption means that no plain text is transmitted at any point during data transmission and the transferred data can only be decrypted by the intended senders and recipients. The sensor data will be transferred in anonymised format by allocating it to the ID of the wearable. Personal data (name of the resident, sex, date of birth, assigned wearables, nursing facility, room information, assigned ward mobile phone/nursing staff) is also stored in a particular part of the cloud backend, the Business Logic Server, and the sensor data will be allocated to the participant’s personal data using the wearable ID. This allocation is essential because otherwise a detected incontinence event cannot be matched to a resident and the responsible nursing staff cannot be informed via digital end device. The merging of sensor data and personal data in the cloud backend meets high security standards and thus ensures that the system offers as few attack points as possible for an unauthorized extraction, manipulation or use of personal data, e.g. by hackers.

After completion of the project, the records, transcripts and questionnaires will be sealed, stored for ten years and then destroyed. The contact data necessary to carry out the study is stored separately from the records, transcripts, questionnaires and description of the results. Both data sets will be kept locked. The mandatory data protection information required by law is complied with (2016/679/EU and State Data Protection Act for the State of Mecklenburg-Vorpommern of 22.05.2018).

### Data monitoring and recording

A Data Monitoring Committee (DMC) is not established as there is no blinding and adverse events can be reported directly to the study staff. All aspects of the intervention are part of the conventional nursing care of incontinence. There are no interim analyses planned and all results will be analysed after the data collection is completed.

### Statistical methods

The data material will be analysed using SPSS. The focus of the evaluation will be on descriptive analyses and correlation analyses. Conclusively, the results will be evaluated and presented in a stratified manner. The following methods will be used to answer the research questions: Regression analyses, MANOVA with measurement repetition, correlation analysis, multilevel analysis and chi-squared tests. The results of the qualitative interviews will first be transcribed and then analysed using qualitative contents analysis [52] and MAXQDA software.

### Logistical implementation of the study project

**Fig. 4:**
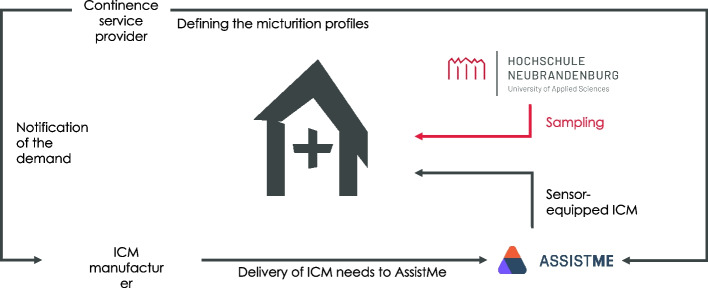
Logistical process of the ICM supply within the study project

In the context of the RCT, in addition to the technical and methodological points, the underlying logistical process must also be comprehensively defined and coordinated with the project partners involved so that regular nursing care is not restricted at any time. Therefore, after randomization of the sample into intervention and control group, detailed micturition profiles of the participating residents are additionally established by the incontinence care provider responsible for the respective nursing facility. The ICM requirements will be reported to the ICM manufacturer. Concerning the intervention group, AssistMe obtains the personal ICM requirements for a usage time period of four to six weeks as well as the ICM from the ICM manufacturer. AssistMe then attaches the digital incontinence sensors to the ICM and sends the material to the according nursing facility. In parallel, the ICM manufacturer delivers the original ICM requirement to the nursing facilities to ensure that the supply is not limited at any point. This will be particularly effective if the RCT has to be paused or ended due to dropout criteria. This will ensure that the standard care can be resumed at any time, independent of the digital incontinence management (see Fig. 4).

### Possible harms

The project partner AssistMe GmbH has already coducted numerous tests of the digital assistance system for incontinence care in the past. Harm due to heat through the sensor or other personally hazardous technical malfunctions can be ruled out.

To ensure that the system and the app function without interference, technical devices with electromagnetic emissions in microwave range are installed within the inpatient care facility (router, charger, node). Damage or incidents due to electromagnetic exposure or compatibility problems are not to be expected, as all necessary electromagnetic safety checks (electromagnetic compatibility) have been carried out and the specifications are fully met by AssistMe GmbH. The tests also showed no restrictions for the participants with regard to freedom of movement or wearing comfort of the ICM. Further comfort tests are currently being carried out to determine whether patients experience unpleasant pressure sensations from the sensor attached to the incontinence material when lying in certain positions (especially in the prone position). However, significant problems are not expected due to the small size and height of the sensor. Regarding the quality of the care, no deterioration is expected since the digital assistance system will only complement traditional nursing measures as part of incontinence care but shall by no means replace them. Standard care is assured for all participants. If problems with the system should develop, those affected in the intervention group can immediately switch to standard care.

In rare cases, participants in both target groups may experience psychological discomfort. On the one hand, this may be due to the fundamental topic of incontinence and incontinence care, which is often still a taboo subject in society. On the other hand, the sensors on the incontinence material record data continuously (24/7), which may result in an unpleasant feeling of being monitored. Particularly in the case of nursing staff, the impression could arise that they may be checked for possible nursing fails. These potential negative perceptions should be countered with transparent information at the beginning of the study as well as with close-meshed discussions with the persons concerned if necessary.

In addition, the secondary target group may also experience a time burden and technical overload, as they are expected to use and apply a new care system in parallel to the standard care of incontinence. Especially at the beginning of the study, when there is no experience in using the assistance system yet, or in already stressful situations in the daily work of the nursing staff, the use of the system could rather be perceived as a burden. These expected impairments are to be countered on the one hand with an intensive explanation of the handling and use of the system, and on the other hand with close-meshed conversations in the case of perceived challenges, especially in the first weeks of the study. Basically, all problems and wishes expressed by both target groups are taken very seriously by the study staff, and they are always responded to promptly.

A further risk could be the occurrence of COVID cases in the care facilities, whereby the facilities or the care personnel may wish to withdraw from participation due to the time required for the study project, or further participation is only possible under more difficult conditions.

Furthermore, there may be unwanted consequences for the study project from an environmental perspective due to the single use of the sensors. As part of the study project, resource-efficient production as well as the possibility of multiple use will be tested. In this regard, the study project aims to find ways to enable a multiple use or sustainable mass production of the incontinence sensors. As part of the project, indications for a more favorable mass production and/or multiple use of the sensor will be developed.

### Audit

All funds and invoices will be managed by study-independent employees in the administration of the University of Applied Sciences Neubrandenburg.

### Distribution

The study results will be made available to the scientific public through conferences and international journals, and to the general public through presentations in healthcare organizations and national congresses. In the long term, the project is intended to contribute to the further development of new quality-assured forms of care and action in nursing practice.

## Discussion

The results of the RCT are expected to provide reliable data regarding improved incontinence care. The focus is primarily on the possible improvement of the quality of life and dignity of those affected, as they are severely impacted by the taboo that often surrounds the issue and by additional health problems associated with incontinence (including falls) [4]. The study results should also show positive effects on the quality of sleep of those affected, which is often very low due to the nature of the condition and the unnecessary changes of incontinence material at night [14]. The use of a sensor-based assistance system for incontinence care could help to significantly reduce the vulnerability of those affected and the use of force when changing the incontinence material [10]. Since progressive urinary and faecal incontinence as well as the associated excessive demands in dealing with it are not uncommon reasons for entering inpatient care, a reliable and easy-to-use digital assistance system could help to relieve the burden on family caregivers and give those affected the opportunity to remain in their homes longer than before [53].

In addition, data will be determined as to whether and how processes in inpatient care settings can be improved using a digital assistance system. In the long term, the use of digital assistance systems for incontinence care may lead to new nursing and care concepts that support inpatient long-term care. The results of the study are to be processed in form of recommendations for action and to provide information on possible transferability to standard care. Focal points are:

- conceptual elaboration concerning nursing facilities with recommendations for data protection
- information of those in need of care
- technical implementation, use and maintenance of the digital assistance system
- cleaning and disposal of the sensor technology
- nursing staff training

Another focus is on economic feasibility studies, which consider implementation and transfer to care settings in inpatient facilities even after the end of the funding period. For outpatient services, the study also provides information on the application and implementation of the digital assistance system.

## Data Availability

The raw datasets can be obtained from the corresponding author upon justified request.

## Abbreviations

BLE: Bluetooth Low Energy
CG: Control group
DMC: Data Monitoring Committee
DNQP: German Network for Quality Development in Nursing
eCRF: Electronic case report forms
GKV-Spitzenverband: German National Association of Statutory Health Insurance Funds
HSNB: University of Applied Sciences Neubrandenburg
ICM: Incontinence material
ID: Identification number
IG: Intervention group
LTE: Long Term Evolution
MMSE: Mini Mental Status Examination
RCT: Randomized controlled trial
SPIRIT 2013 Statement: Defining standard protocol items for clinical trials
SPSS: Statistical Package for the Social Sciences
TA-EG: Questionnaire to measure the affinity for technology scale

## References

[1] Nussbaumer-Streit B, Lohrmann C (2011). Prävalenz der Inkontinenz und Pflegemaßnahmen. ProCare.

[2] Beutel ME, Hessel A, Schwarz R, Braehler E (2005). Prävalenz der Urininkontinenz in der deutschen Bevölkerung. Komorbidität, Lebensqualität, Einflussgrössen [Prevalence of urinary incontinence in the German population]. Urologe A.

[3] Enck P (1994). Epidemiologie der Stuhlinkontinenz. Kontinenz.

[4] Suzuki M, Miyazaki H, Kamei J (2019). Ultrasound-assisted prompted voiding care for managing urinary incontinence in nursing homes: a randomized clinical trial. Neurourol Urodyn.

[5] Musa MK, Saga S, Blekken LE, Harris R, Goodman C, Norton C (2019). The prevalence, incidence, and correlates of fecal incontinence among older people residing in care homes: a systematic review. J Am Med Dir Assoc.

[6] Suhr R, Lahmann NA (2018). Urinary incontinence in home care: a representative multicenter study on prevalence, severity, impact on quality of life, and risk factors. Aging Clin Exp Res.

[7] Chiarelli PE, Mackenzie LA, Osmotherly PG (2009). Urinary incontinence is associated with an increase in falls: a systematic review. Aust J Physiother.

[8] Xu D, Kane RL (2013). Effect of urinary incontinence on older nursing home residents' self-reported quality of life. J Am Geriatr Soc.

[9] Hayder D, Schnepp W (2010). Umgang mit Harninkontinenz: Ergebnisse einer qualitativen Studie mit Betroffenen und pflegenden Angehörigen. Pflege.

[10] Schlueter W, Oleksiw K, Rosenbaum U, Herold C, Lindner P. *Würde und Inkontinenz. Forschungsbericht zum Projekt „Bestimmung von Faktoren für eine würdevolle Inkontinenzversorgung von Bewohnern in Altenpflegeeinrichtungen“*. Zwickau: Westsächsische Hochschule. 2010

[11] DNQP – Deutsches Netzwerk für Qualitätsentwicklung in der Pflege, ed. *Expertenstandard. Förderung der Harninkontinenz in der Pflege.* 1^st^ Update 2014. March 2014*.* Hochschule Osnabrück.

[12] Veronese N, Soysal P, Stubbs B (2018). Association between urinary incontinence and frailty: a systematic review and meta-analysis. Eur Geriatr Med.

[13] Yang E, Lisha NE, Walter L, Obedin-Maliver J, Huang AJ (2018). Urinary Incontinence in a national cohort of older women: implications for caregiving and care dependence. J Womens Health (Larchmt).

[14] da Luz Verônica D, Fank F, Pereira FDS, Mazo GZ (2022). Sleep quality and urinary incontinence in elderly female exercise practitioners. Sleep Sci.

[15] von Graf Schulenberg JM (2007). Kosten der Harninkontinenz in Deutschland. Gesundheitsökonomie Qualitätsmanagement.

[16] Moore KN, Fader M, Getliffe K (2007). Long-term bladder management by intermittent catheterisation in adults and children. Cochrane Database Syst Rev.

[17] Wulf T. Welche ökonomischen Dimensionen hat Inkontinenz? In: Parabo F, Mueller SC. *Inkontinenz Fragen und Antworten*. Deutscher Ärzte-Verlag Köln GmbH 2009

[18] Neubauer G, Stiefelmeyer S. (2005). Economic Costs of Urinary Incontinence in Germany. In: Becker HD, Stenzl A, Wallwiener D, Zittel, TT (eds). *Urinary and Fecal Incontinence. An interdisziplinary Approach*. Springer Berlin Heidelberg. 2005:25–31. 10.1007/3-540-27494-4_3

[19] Seers K (2018). Facilitating Implementation of Research Evidence (FIRE): An international cluster randomised controlled trial to evaluate two models of facilitation informed by the Promoting Action on Research Implementation in Health Services (PARIHS) framework. Implement Sci.

[20] King AJ, Evans M, Moore TH (2015). Prostate cancer and supportive care: a systematic review and qualitative synthesis of men's experiences and unmet needs. Eur J Cancer Care (Engl).

[21] Seo HJ, Lee NR, Son SK, Kim DK, Rha KH, Lee SH (2016). Comparison of robot-assisted radical prostatectomy and open radical prostatectomy outcomes: a systematic review and meta-analysis. Yonsei Med J.

[22] Thomas LH, Coupe J, Cross LD, Tan AL, Watkins CL (2019). Interventions for treating urinary incontinence after stroke in adults. Cochrane Database Syst Rev.

[23] Lopes-Souza P, Dionello CF, Sá-Caputo DDC (2018). Whole body vibration exercise in the management of cancer therapy-related morbidities: a systematic review. Drug Discov Ther.

[24] Sayılan AA, Özbaş A (2018). The effect of pelvic floor muscle training on incontinence problems after radical prostatectomy. Am J Mens Health.

[25] Sussman RD, Richter LA, Tefera E (2016). Utilizing technology in assessment of lower urinary tract symptoms: a randomized trial of electronic versus paper voiding diaries. Female Pelvic Med Reconstr Surg.

[26] Hoffman V, Soederstroem L, Samuelsson E (2017). Self-management of stress urinary incontinence via a mobile app: two-year follow-up of a randomized controlled trial. Acta Obstet Gynecol Scand.

[27] Cho JH, Choi JY, Kim NH (2021). A smart diaper system using bluetooth and smartphones to automatically detect urination and volume of voiding: prospective observational pilot study in an acute care hospital. J Med Internet Res.

[28] Raepsaet C, Serraes B, Verhaeghe S, Beeckman D (2021). Integrating sensor technology in disposable body-worn absorbent products: a qualitative study to define user profile, (Technical) criteria, conditions, and potential benefits. J Wound Ostomy Continence Nurs.

[29] Ernste P (2016). Moderne Technik für ein selbstbestimmtes Leben im Alter – Was denkt die Zielgruppe?. Forschung Aktuell.

[30] Chan AW, Tetzlaff JM, Altman DG (2013). SPIRIT 2013 statement: defining standard protocol items for clinical trials. Ann Intern Med.

[31] Cochrane Deutschland, Institut für Medizinische Biometrie und Statistik Freiburg, Arbeitsgemeinschaft der Wissenschaftlichen Medizinischen Fachgesellschaften- Institut für Medizinisches Wissensmanagement, Ärztliches Zentrum für Qualität in der Medizin. *Manual zur Bewertung des Biasrisikos in Interventionsstudien*. 2^nd^ ed. 2021. Available at Cochrane Deutschland: https://www.cochrane.de/de/literaturbewertung

[32] ICH-E9 (2006). Statistical principles for clinical trials. Note for guidance on statistical principles for clincal trials.

[33] Kernan WN, Viscoli CM, Makuch RW, Brass LM, Horwitz RI (1999). Stratified randomization for clinical trials. J Clin Epidemiol.

[34] Muz MS, Weigl B, Schmidt S, Dibelius O, Offermanns P, Schmidt S (2016). Studien zur Schmerzerfassung und Sterbebegleitung bei Menschen mit Demenz. Palliative Care für Menschen mit Demenz.

[35] Lukas A, Niederecker T, Günther I, Mayer B, Nikolaus T (2013). Self- and proxy report for the assessment of pain in patients with and without cognitive impairment: experiences gained in a geriatric hospital. Z Gerontol Geriatr.

[36] Folstein MF, Folstein SE, McHugh PR (1975). "Mini-mental state". A practical method for grading the cognitive state of patients for the clinician. J Psychiatr Res.

[37] Hoegel J. Stratifizierung. In: Lenk C, Duttge G, Fangerau H. *Handbuch Ethik und Recht der Forschung am Menschen*. Springer-Verlag Berlin Heidelberg 2014;653–656. 10.1007/978-3-642-35099-3

[38] Kruse J. *Reader „Einführung in die qualitative Interviewforschung“.* Überarbeitete, korrigierte und umfassend ergänzte Version. Freiburg. 2011

[39] Oliver D, Britton M, Seed P, Martin FC, Hopper AH (1997). Development and evaluation of evidence-based risk assessment tool (STRATIFY) to predict which elderly inpatients will fall: case-control and cohort studies. BMJ.

[40] Oliver D, Daly F, Martin FC, McMurdo ME (2004). Risk factors and risk assessment tools for falls in hospital in-patients: a systematic review. Age Ageing.

[41] Tinetti ME (1986). Performance-oriented assessment of mobility problems in elderly patients. J Am Geriatr Soc.

[42] Koepcke S, Meyer G, Mahler C (2011). Sturzrisikoassessment. Reuschenbach B.

[43] Holzhausen M, Kuhlmey A, Martus P (2010). Individualized measurement of quality of life in older adults: development and pilot testing of a new tool. Eur J Ageing.

[44] Akerstedt T, Gillberg M (1990). Subjective and objective sleepiness in the active individual. Int J Neurosci.

[45] Niederl, T. Untersuchungen zu kumulativen psychischen und physiologischen Effekten des fliegenden Personals auf der Kurzstrecke: Am Beispiel des Flugbetriebes der Boeing 737 Flotte der Deutschen Lufthansa AG. Forschungsbericht 2007–17. Köln. Deutsches Zentrum für Luft- und Raumfahrt, Institut für Luft- und Raumfahrtmedizin. 2007

[46] Bloch KE, Schoch OD, Zhang JN, Russi EW (1999). German version of the Epworth sleepiness scale. Respiration.

[47] Hays RD, Stewart AL, Stewart AL, Ware JE (1992). Sleep measures. Measuring functioning and well-being: The Medical Outcomes Study approach.

[48] Hedges C (2005). Sleep, memory, and learning in off-pump coronary artery bypass patients. Res Nurs Health.

[49] Johns MW (1994). Sleepiness in different situations measured by the Epworth sleepiness scale. Sleep.

[50] Rand Health. *MOS Sleep Scale Survey Instrument*. 2017. www.rand.org/health/surveys_tools/mos /mos_sleep.html (Zugriff: 10.09.20).

[51] Chittrakul J, Siviroj P, Sungkarat S, Sapbamrer R (2020). Multi-system physical exercise intervention for fall prevention and quality of life in pre-frail older adults: a randomized controlled trial. J Environ Res Public Health.

[52] Mayring P (2015). Qualitative Inhaltsanalyse: Grundlagen und Techniken.

[53] Messer M (2012). Wie erleben pflegende Angehörige die Inkontinenz ihres an Alzheimer-Demenzerkrankten Ehepartners?: Das Erleben von Inkontinenz aus Sicht pflegender Ehepartner. HeilberufeSCIENCE.

